# The dynamical study of O(^1^D) + HCl(v = 0, j = 0) reaction at hyperthermal collision energies

**DOI:** 10.1186/1752-153X-7-177

**Published:** 2013-11-15

**Authors:** Meihua Ge, Huan Yang, Yujun Zheng

**Affiliations:** 1 School of Physics, Shandong University, Jinan 250100, China

**Keywords:** O(^1^D) + HCl, Hyperthermal, Stereodynamics, Alignment, Orientation

## Abstract

**Backgrounds:**

The quasi-classical trajectory calculations for O(^1^D) + HCl → OH + Cl (R1) and O(^1^D) + HCl → ClO + H (R2) reactions have been performed at hyperthermal collision energies (60.0, 90.0, and 120.0 kal/mol) on the ^1^A' state. Reaction probabilities and integral cross sections are calculated. The product rotational distributions for the two channels, and the product rotational alignment parameters are investigated. Also, the alignment and the orientation of the products have been predicted through the angular distribution functions (concerning the initial/final velocity vector, and the product rotational angular momentum vector). To have a deeper understanding of the natures of the vector correlation between reagent and product relative velocities, a natural generalization of the differential cross section __PDDCS_00_, is calculated.

**Results:**

The OH + Cl channel is the main product channel and is observed to have essentially isotropic rotational distributions. The ClO + H channel is found to be clearly rotationally polarized.

**Conclusions:**

The dynamical, especially the stereodynamical characters are quite different for the two channels of the title reaction. Most reactions occur directly, except for R2 reaction at the collision energies of 60.0 and 120.0 kcal/mol. The alignment and orientation effects are weak/strong for R1/R2 reaction. The well structure on the potential energy surface and hyperthermal collision energies might result in the dynamical effects.

## Background

Considerable attention has been devoted to the O(^1^D) + HCl reaction [1-22], due in part, to its significant role in stratospheric chemistry. Using *ab initio* self consistant field (SCF) and configuration interaction (CI) methods, Bruna *et al*. [1] reported potential curves for the ground and various valence and Rydberg excited states of HOCl and HClO. The angular and velocity distributions of ClO product from the reaction of O(^1^D) + HCl at 12.2 kcal/mol collision energy were calculated in a crossed-molecular-beam study in Ref. [2]. Experimentally, the reactions of O(^1^D) + HCl → OH + Cl and OCl + H were studied at an average collision energy of 7.6, 7.7, and 8.8 kcal/mol through the resonance-enhanced multiphoton ionization technique [3]. Nascent state-resolved ClO (X^2^П) radicals produced in reaction of O(^1^D) with HCl were measured by employing the technique of vacuum-ultraviolet laser-induced fluorescence [4]. Hernandez *et al*. [5] calculated the potential energy surface (PES) of the O(^1^D) + HCl reaction and performed a quasi-classical trajectory (QCT) study on this PES. Cross sections over the collision energy range of 0.0-20.0 kcal/mol were presented and product angular distributions were given at the collision energies of 7.6 and 12.2 kcal/mol. Alvariño *et al*. [6] studied the dependence of calculated product rotational polarization on the scattering angle for the title reaction using QCT method. An accurate *ab initio* HOCl PES was constructed by Skokov *et al*. [7] in 1998. Through a QCT calculation [8], the product angular distribution and dihedral angle distribution for the ClO forming process are performed together with product vibrational distribution for the OH forming process. The quantum and QCT reaction probabilities (RPs) [9] were presented over the collision energy range of 2.3-18.4 kcal/mol by Christoffel *et al*.. At the collision energy of 12.2 kcal/mol, integral cross sections (ICSs) for vibrational states summed over rotational states for the ClO and OH products, and translational energy distributions of the ClO product were also performed [9]. Based on Ref. [7], a global PES was constructed for the X^1^A' electronic ground state of HOCl including the accurate HClO isomer [10]. Vibrational energy levels and intensities were computed for both HOCl and HClO up to the OH + Cl dissociation limit and above the isomerization barrier using the PES of Ref. [10]. Bittererová *et al*. [11] performed a wave-packet calculation to study the effect of reactant rotation and alignment on product branching in the O(^1^D) + HCl → ClO + H, OH + Cl reactions using the PES of Ref. [10]. A new fit to extensive *ab initio* calculations of a global potential [10] and the quantum wave packet calculations of the O(^1^D) + HCl → ClO + H, OH + Cl reactions were reported by Bittererová *et al*. [12]. Accurate time-dependent wavepacket calculation for the O(^1^D) + HCl reaction was carried out by Lin *et al*. [13]. Recently, we have studied the effects of the collision energy and reagent vibrational excitation on the reaction of O(^1^D) + HCl → OH + Cl [22].

However, most of these studies were focused on the case of low collision energies. As well known, hyperthermal collisions act a part in the chemistry of extreme environments, such as those encountered in plasma, rocket plumes, and space vehicles in low-earth orbit. The hyperthermal O + HCl chemistry plays an important role in the reacting flows coming from the interaction of a jet and the rarefied atmosphere [23], and we need the data of accurate reaction cross sections and branching ratios at high collision energies to assess its importance. The dynamics of high-energy collisions remains mostly unexplored, and there are only a few studies concerned with the O(^3^P) + HCl reaction [24,25].

The title reaction is especially demanding, and interesting, due to the presence of two product channels,

(R1) $$O\left({}^{1}D\right)+\mathrm{HCl}\left({}^{1}\Sigma^{+}\right)\to \mathrm{OH}\left({}^{2}П\right)+\mathrm{Cl}\left({}^{2}P\right)$$

(R2) $$\to \mathrm{ClO}\left({}^{2}П\right)+H\left({}^{2}S\right)$$

As noted in Ref. [12], when the collision energy is below 0.55 eV (12.68 kcal/mol), the quantum integral cross sections (ICSs) display an inverse dependence on the collision energy, and the OH product is favoured over the ClO product. But what will happen when the collision energy is hyperthermal?

In this paper, based on the recent-developed ^1^A' PES [12], a quasi-classical trajectory (QCT) calculation is performed on the O(^1^D) + HCl(v = 0, j = 0) reaction so as to study the dynamical, especially the stereodynamical characteristics at hyperthermal collision energies. To evaluate the importance of the hyperthermal O + HCl chemistry, RPs, cross sections and branching ratios at high collision energies are investigated. Also, our investigation can provide necessary data to the hyperthermal O + HCl chemistry. The products for R1 and R2 reactions have hot rotational populations. Alignment and orientation effects are shown through two angular distribution functions. The scattering directions of the products are also studied through the PDDCS_00_ results. The statistical errors are marked as error bars in the Figures.

### Methodology and computational details

In the framework of quasi-classical trajectory (QCT) approach [21,22,26-34], the center-of-mass (CM) frame is used. The reagent relative velocity vector ***k*** is chosen to be parallel to the *z* axis, and the scattering plane contains the initial and final velocity vectors (noted as ***k*** and ***k***', respectively). In the CM frame, *θ*_*r*_ and *φ*_*r*_ are the corresponding polar and azimuthal angles of the product rotational momentum ***j***', respectively. The scattering angle between ***k*** and ***k***' is marked as *θ*_*t*_, namely

(1) $$\cos\theta_{t}=\frac{\left(k\cdot k'\right)}{\left(\left|k\right|\cdot \left|k'\right|\right)}.$$

The numbers of reactive trajectory and total trajectory are marked as *N*_*r*_ and *N*_*tot*_ in due order. The RP can be expressed as

(2) $$P=\frac{N_{r}}{N_{\mathrm{tot}}}.$$

The ICS *σ* can be defined as

(3) $$\sigma =\pi b_{\max}^{2}\frac{N_{r}}{N_{\mathrm{tot}}},$$

where *b*_max_ denotes the maximum value of the impact parameter *b*.

The associated uncertainties with the ICS can be calculated according to *Δσ* = [(*N*_tot_ − *N*_r_)/(*N*_tot_ · *N*_r_)]^1/2^*σ*.

The differential cross section (DCS) is given by

(4) $$\frac{d\sigma }{d\left(\cos\theta_{t}\right)}=\frac{N_{r}\left(\theta_{t}\right)\cdot \pi b_{\max}^{2}}{2\pi \sin\theta_{t}\cdot N_{\mathrm{tot}}}.$$

During reactive encounter, the total angular momentum is conserved [26]

(5) j+l=j'+l',

here ***l*** and ***l***' are the reagent and product orbital momenta, respectively. When ***j*** is small (as is common), the rotation of the product can only result from ***l***. The distribution of ***j***' is described by *P*(*θ*_*r*_), which can be expanded by a set of Legendre polynomials

(6) $$P\left(\theta_{r}\right)=\frac{1}{2}\sum_{k}\left(2k+1\right)a_{0}\left(k\right)P_{k}\left(\cos\theta_{r}\right).$$

Here the polarization parameter *a*_0_(*k*) can be expressed as

(7) $$\begin{array}{l} a_{0}\left(k\right)=\int_{0}^{\pi }P\left(\theta_{r}\right)P_{k}\left(\cos\theta_{r}\right)\sin\theta_{r}d\theta_{r}\\ \;=\langle P_{k}\left(\cos\theta_{r}\right)\rangle . \end{array}$$

*a*_0_(2) indicates the product rotational alignment

(8) $$a_{0}\left(2\right)=\langle P_{2}\left(\cos\theta_{r}\right)\rangle =\frac{1}{2}\langle 3\cos^{2}\theta_{r}‒1\rangle .$$

*P*(*φ*_*r*_) denotes the dihedral angle distribution, which can be expanded in the Fourier series

(9) $$P\left(\varphi_{r}\right)=\frac{1}{2\pi }\left[1+\sum_{\mathrm{even},n\geq 2}a_{n}\cos\left(n\varphi_{r}\right)+\sum_{\mathrm{odd},n\geq 1}b_{n}\sin\left(n\varphi_{r}\right)\right].$$

The expansion coefficients *a*_*n*_ and *b*_*n*_ are given by

(10) $$a_{n}=2\langle \cos n\varphi_{r}\rangle ,$$

(11) $$b_{n}=2\langle \sin n\varphi_{r}\rangle .$$

The full three-dimensional angular distribution associated with ***k*-*k***'**-*j***' correlation can be expressed as

(12) $$P\left(\omega_{t},\omega_{r}\right)=\sum_{\mathrm{kq}}\frac{2k+1}{4\pi }\frac{1}{\sigma }\frac{d\sigma_{\mathrm{kq}}}{d\omega_{t}}C_{\mathrm{kq}}{\left(\theta_{r},\varphi_{r}\right)}^{*},$$

where *C*_*kq*_(*θ*_*r*_, *φ*_*r*_) are the modified spherical harmonics and the angles *ω*_*t*_ = *θ*_*t*_, *φ*_*t*_ and *ω*_*r*_ = *θ*_*r*_, *φ*_*r*_ refer to the coordinates of the unit vectors ***k***' and ***j***' along the directions of the product relative velocity and rotational angular momentum vectors, respectively. $\frac{1}{\sigma }\frac{d\sigma_{\mathrm{kq}}}{d\omega_{t}}$ is a generalized polarization-dependent differential cross section (PDDCS), and it can be written as

(13) $$\frac{1}{\sigma }\frac{d{\sigma_{\mathrm{kq}}}_{\pm }}{d\omega_{t}}=\sum_{k_{1}}\frac{2k_{1}+1}{4\pi }S_{\mathrm{kq}\pm }^{k_{1}}C_{k_{1}-q}\left(\theta_{t},0\right).$$

Here the expectation value $S_{\mathrm{kq}\pm }^{k_{1}}$ is given by

(14) $$S_{\mathrm{kq}\pm }^{k_{1}}=\langle C_{k_{1}q}\left(\theta_{t},0\right)C_{\mathrm{kq}}\left(\theta_{r},0\right)\left[{\left(-1\right)}^{q}e^{\mathrm{iq}\varphi_{r}}\pm e^{-\mathrm{iq}\varphi_{r}}\right]\rangle .$$

The angular brackets 〈⋯〉 in Eq. (14) represent the average over all angles.

The initial ro-vibrational quantum numbers of the HCl reactant are set as v = 0, and j = 0. 1,000,000 trajectories are used on the ^1^A' electronic states at the collision energies of 60.0, 90.0 and 120.0 kcal/mol. The time integral step size is 10^-4^ ps. The maximum values of impact parameter *b*_max_ are 2.80/1.15 (60.0 kcal/mol), 2.81/1.55 (90.0 kcal/mol), and 2.86/1.05 (120.0 kcal/mol) for R1/R2 reaction and the unit is in Angstrom.

**Figure 1:**
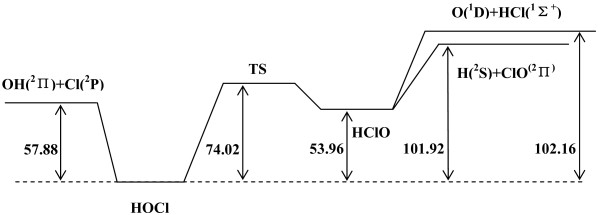
**The schematic of the energetics (in kcal/mol) of the O(**^**1**^**D) + HCl *ab initio *** **global potential.**

The PES we used is constructed by Bittererová *et al.*[12]. The title reaction proceeds without a barrier to either set of products, but via two complex regions, HOCl and HClO. For R1 (R2) reaction, according to Ref. [12], ^1^A' state has a deep well in bent geometry corresponding to stable HOCl (HClO) molecule and the well depth is -102.16 (-48.20) kcal/mol. The schematic of the energetics of the O(^1^D) + HCl *ab initio* global potential is exhibited in Figure 1.

**Figure 2:**
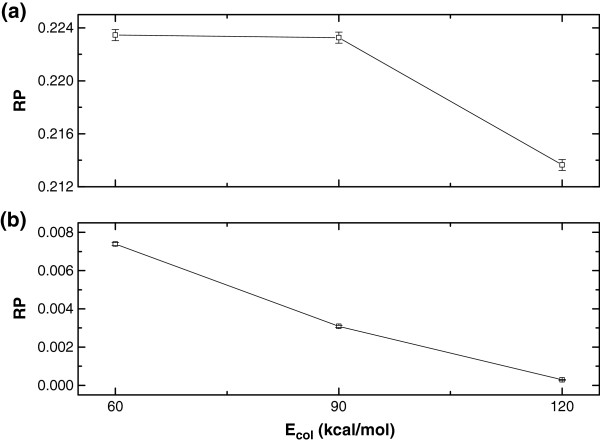
RPs at the collision energies of 60.0, 90.0 and 120.0 kcal/mol for (a) O + HCl → OH + Cl (R1), and (b) O + HCl → ClO + H (R2) reactions.

For the A + BC → AB + C reaction, in the impulse model [27], the product rotational angular momentum ***j***' could be described with the reagent orbital and rotational angular momenta, ***l*** and ***j***, *i.e.*$j'=l\sin^{2}\beta +j\cos^{2}\beta +J_{1}\frac{m_{B}}{m_{\mathrm{AB}}}$, where $J_{1}=\sqrt{\mu_{\mathrm{BC}}R}\left(r_{\mathrm{AB}}\times r_{\mathrm{CB}}\right)$ and $\cos^{2}\beta =\frac{m_{A}m_{C}}{\left(m_{A}+m_{B}\right)\left(m_{B}+m_{C}\right)}$. *μ*_*BC*_ is the reduced mass of the BC molecule, *R*, the repulsive energy, and *r*_*AB*_, ***r***_C*B*_, the unit vectors where B points to A and where B points to C, respectively, *β* is known as the skew angle. For R1 (R2) reaction, *β* ≈ 17*°* (*β* ≈ 85*°*), which is a pretty small (large) angle. Larger polarization properties are expected for the products with the smaller value of cos^2^*β* ( *i.e.* R2 reaction) according to the kinematic limit, which could be observed via the alignment parameters. ***l*** sin ^**2**^*β* + j cos ^**2**^*β* is symmetric, which leads to the symmetric distribution of *P*(*θ*_*r*_) in Figure 2. However, $\frac{J_{1}m_{B}}{m_{\mathrm{AB}}}$ shows a preferred direction due to the repulsive energy and results in the biased orientation of the products as shown in *P*(*φ*_*r*_) distributions.

## Results and discussion

The results for R1 and R2 reactions are found to be quite different due to the different dynamical reaction channels, which can also be observed in the two (HF and DF) product channels of F + HD reaction [35].

### RPs and ICSs

**Figure 3:**
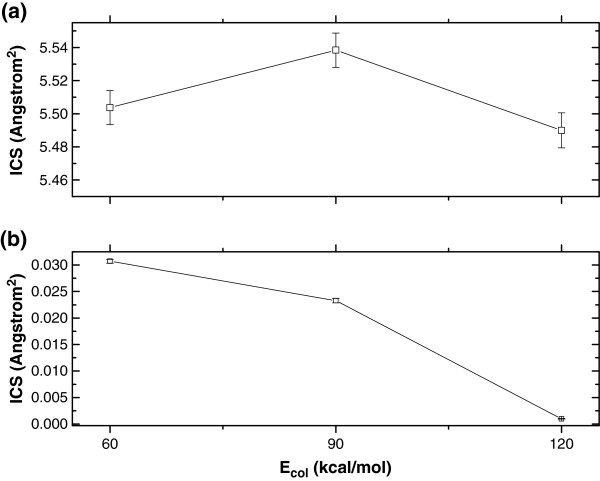
ICSs at the collision energies of 60.0, 90.0 and 120.0 kcal/mol for (a) O + HCl → OH + Cl (R1), and (b) O + HCl → ClO + H (R2) reactions.

Figures 2 and 3 show the results of RPs and ICSs at the collision energies of 60.0, 90.0, and 120.0 kcal/mol. The OH products shown in Figure 2(a) and Figure 3(a) are apparently favoured over the ClO products shown in Figure 2(b) and Figure 3(b), which agrees well with the quantum results at low collision energies [12]. With the increase of the collision energy, both RP and ICS obviously decrease except for ICS at the collision energy of 90.0 kcal/mol for R1 reaction. The title reaction proceeds without a potential barrier and typically by complex formation, which can be displayed through the quantum RPs [12]. This oscillating structure might cause the translational excitation to impede the reaction. Also, the quantum ICSs [12] show the inverse dependence on the initial relative kinetic energy, and the OH product is favoured over the ClO product. The statistical errors for RPs are ± 0.00042/ ± 0.000086, ± 0.00042/ ± 0.000055, ± 0.00041/ ± 0.000017 for R1/R2 reaction at E_col_ = 60.0, 90.0 and 120.0 kcal/mol, respectively.

The branching ratios $\frac{\sigma_{\mathrm{ClO}}}{\sigma_{\mathrm{OH}}}$ are about 0.0056(± 0.000075), 0.0042(± 0.000084) and 0.0002(± 0.000011) at the collision energies of 60.0, 90.0 and 120.0 kcal/mol in due order. It is obvious that the branching ratio rapidly decreases with the increase in the collision energy. The branching ratios are much smaller than those at the lower collision energies [2,3,12,14].

### The product rotational distributions (PRDs)

**Figure 4:**
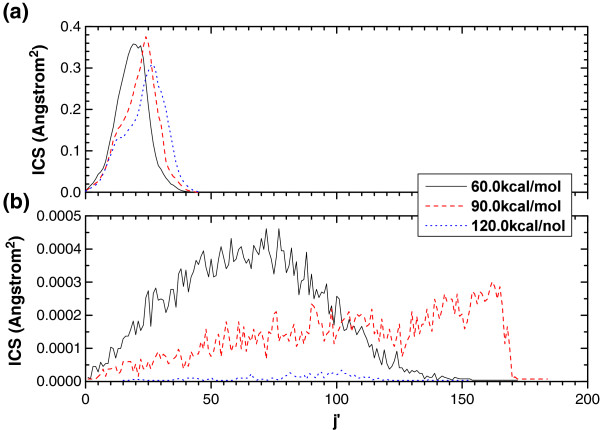
PRDs for (a) O + HCl → OH + Cl (R1), and (b) O + HCl → ClO + H (R2) reactions.

Figure 4 shows the PRDs of R1 (as shown in Panel (a)) and R2 (as shown in Panel (b)) reactions. Obviously, the products for R1 and R2 reactions are rotationally hot. The peak shifts towards larger rotational quantum numbers except for R2 reaction at the collision energy of 120.0 kcal/mol. The PRDs of R2 reaction show much broader ranges than those of R1 reaction due to larger polarization properties for R2 as mentioned above. For the Li + HF → Li + F reaction (which also belongs to Heavy heavy-Light (HHL) scheme), LiF product is also produced in highly excited states [35,36].

As mentioned in Refs. [35-37], vector correlation of angular momentum orientation and alignment in chemical reactions can provide rich information on the reaction dynamics. By analyzing the alignment parameters, two angular distributions (*P*(*θ*_*r*_) and *P*(*φ*_*r*_)), and PDDCS_00_, we can get a view of the stereodynamical information of the title reaction.

### The product rotational alignment

**Figure 5:**
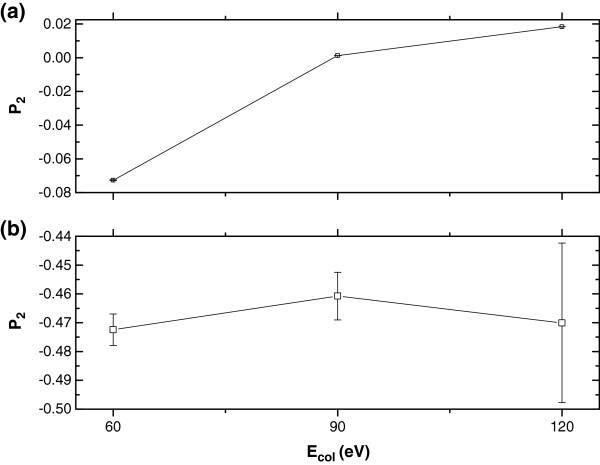
***P***_**2 **_**values at the collision energies of 60.0, 90.0 and 120.0 kcal/mol for (a) O + HCl → OH + Cl (R1), and (b) O + HCl → ClO + H (R2) reactions.**

For R1 reaction as shown in Figure 5(a), *P*_2_ slightly increases with the increase of the collision energy. The value of *P*_2_ becomes less negative (-0.073, 0.001, and 0.018 for E_col_ = 60.0, 90.0 and 120.0 kcal/mol, respectively) and the product rotational angular momentum tends to have a less anisotropic distribution. With the increase of the collision energy, the degree of alignment is almost invariant (only slightly increases) which could be ascribed to the Heavy Light-heavy (HLH) mass combination [6]: much angular momentum transfers from the reagent orbit to the product orbit, ***l***-***l***', and little to rotation. Therefore, large variations in the direction of ***j***' are needed for compensating even small variations in ***l***'.

For R2 reaction, the *P*_2_ values at the three collision energies are approaching to -0.5, which indicates that the product rotation strongly aligned perpendicular to the reagents’ relative velocity ***k***. This is a typical feature of the HHL system [6,26,35,36]. A significant feature of this kind of reaction is the kinematic limit, *i.e.*, the initial orbital angular momentum ***l*** completely goes into the product rotational angular momentum ***j***', which leads to the alignment character. This is consistent with the alignment of Li + HF → LiF + H [35,36]. The significant disposal of angular momentum in the product orbital motion, ***l*** → ***j***' + ***l***' declines the product rotational alignment, particularly at lower collision energies, so the calculated *P*_2_ values deviate slightly from -0.5, which could also be supported by the *P*(*θ*_*r*_) results.

According to the results of H^+^+D_2_(v = 0, j = 0) → HD(v’ , j’) + D^+^ reaction [37], the alignment of rotational angular momentum of HD products is nearly always close to zero due to the long-lived resonances. However, H^+^+D_2_(v = 0, j = 0) → HD(v’ , j’) + D^+^ reaction belongs to Light light-light (LLL) scheme, and in the reference [37] the alignment is state-resolved, however, our results are the sum of all states. So there are different results for the alignment parameters. And through the observation of three internuclear distances with the propagation of collision time, we can find that the reactions proceed directly except for the collision energies of 60.0 and 120.0 kcal/mol for R2 reaction.

### *P*(*θ*_*r*_) distributions

*P*(*θ*_*r*_) is the distribution function reflecting the ***k***-***j*'** correlation, which is sensitive to two factors: the characters of PES and the mass factor. Obviously, there is a discrepancy between *P*(*θ*_*r*_) distributions of the two reactions due to the different mass factors of the two reactions. For R1 and R2 reactions, at all the collision energies, the *P*(*θ*_*r*_) distribution functions are symmetric with respect to *θ*_*r*_ = 90*°* as analyzed above. The alignment effects are obscure /obvious for R1/R2 reaction, which is supported by *P*(*φ*_*r*_) distributions in Figure 7.

**Figure 6:**
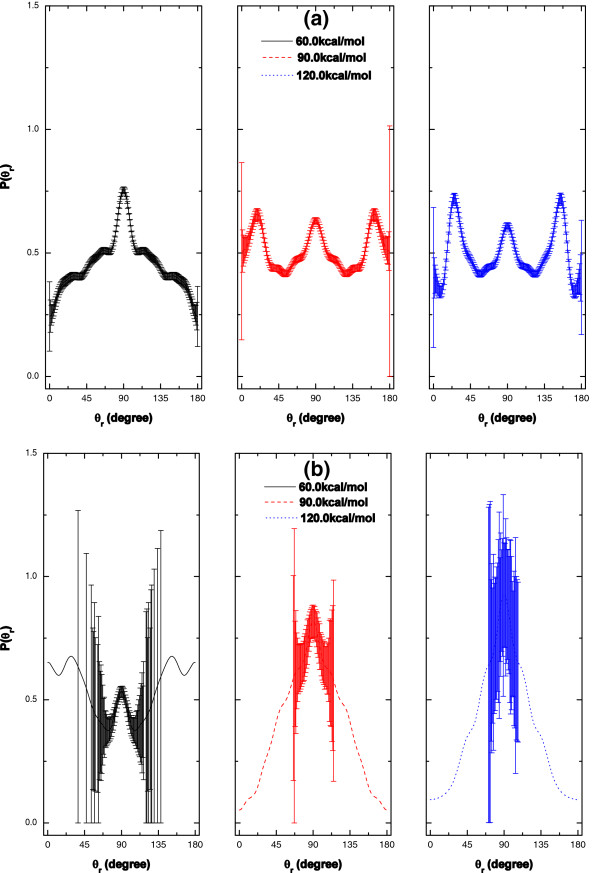
***P*** **(*φ*** **r) distributions at the collision energies of 60.0, 90.0 and 120.0 kcal/mol for (a) O + HCl → OH + Cl (R1), and (b) O + HCl → ClO + H (R2) reactions.**

For R1 reaction shown in Panel (a), the largest peak appears at *θ*_*r*_ = 90*°* at E_col_ = 60.0 kcal/mol. At the collision energy of 90.0 kcal/mol, the largest peaks are around *θ*_*r*_ = 90*°*, 20° and 160°. Around *θ*_*r*_ = 26*°* and 154°, there are the largest two peaks at E_col_ = 120.0 kcal/mol.

**Figure 7:**
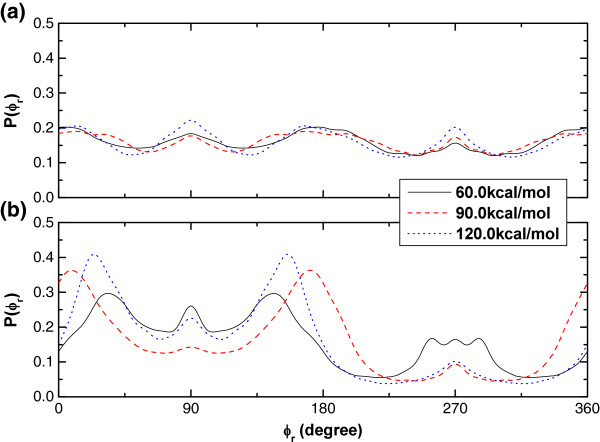
***P*** **(*θ*** **r) Distributions at the collision energies of 60.0, 90.0 and 120.0 kcal/mol for (a) O + HCl → OH + Cl (R1), and (b) O + HCl → ClO + H (R2) reactions.**

For R2 reaction shown in Panel (b), it is a HHL system, with the mass factor – $\cos^{2}\beta =\frac{m_{O}m_{H}}{\left(m_{O}+m_{\mathrm{Cl}}\right)\left(m_{\mathrm{Cl}}+m_{H}\right)}\approx 0.0086$ approaching to zero, which manifests that the product rotational alignment is strong with regard to the initial velocity vector ***k***[26], which could be also observed through the alignment parameter in Figure 5(b) and *P*(*φ*_*r*_) distributions in Figure 7(b).

### *P*(*φ*_*r*_) distributions

*P*(*φ*_*r*_) describes the ***k***-***k***'-***j***' correlation, which reflects the polarization of product rotational angular momentum ***j***'. The *P*(*φ*_*r*_) distribution is asymmetric with respect to the ***k***-***k***' scattering plane (*i.e.* at about *φ*_*r*_ = 180*°*), which could be explained by the impulse model [27] as mentioned above.

For R1 reaction in Figure 7(a), there are only several small peaks, implying that the orientation effects are not obvious at the three different collision energies, which agrees well with the alignment parameter in Figure 5(a) and *P*(*θ*_*r*_) distributions in Figure 6(a).

For R2 reaction in Figure 7(b), the largest peaks appear around *φ*_*r*_ = 0*°* (or 360°)/ *φ*_*r*_ = 180*°*, implying that the ClO molecular axis vector orients along *x*/–*x* axis.

### PDDCS_00_

PDDCS_00_ is proportional to DCS, which can be used to describe the ***k***-***k***' correlation. Through PDDCS_00_, we can study the scattering direction of the product.

**Figure 8:**
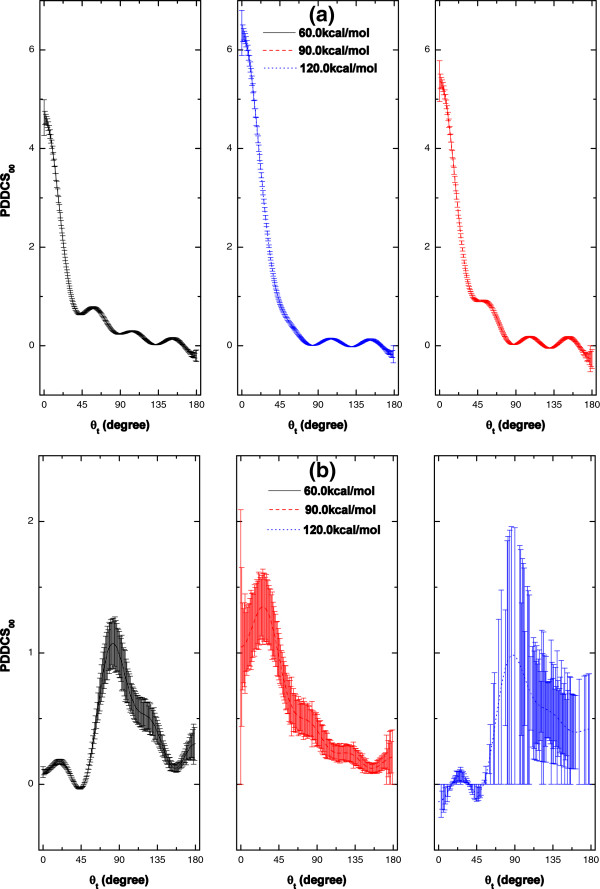
**PDDCS**_**00 **_**as a function of scattering angle at the collision energies of 60.0, 90.0 and 120.0 kcal/mol for (a) O + HCl → OH + Cl (R1), and (b) O + HCl → ClO + H (R2) reactions.**

**Figure 9:**
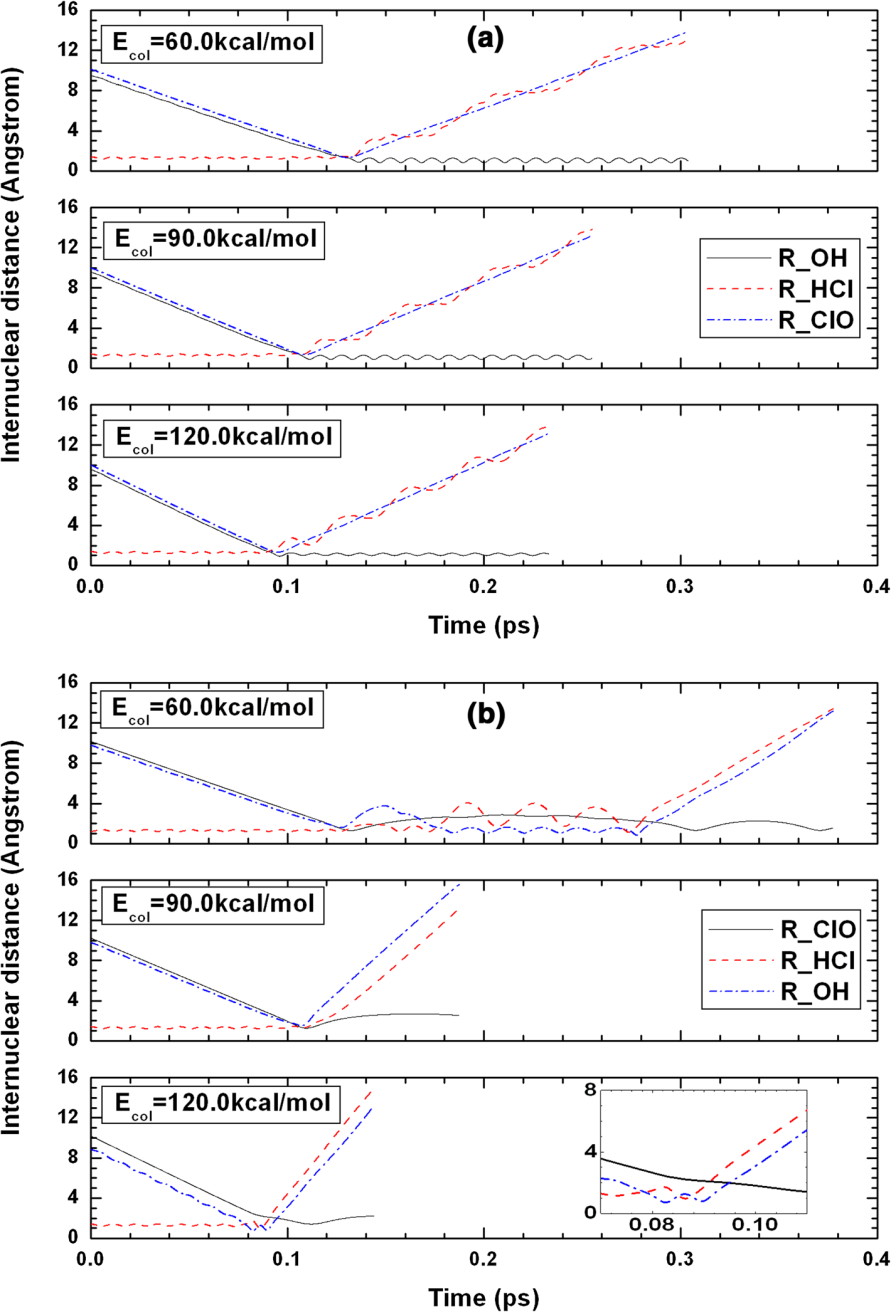
Internuclear distances of OH, HCl and ClO (marked as R_OH, R_HCl and R_ClO, respectively) as a function of propagation time at 60.0, 90.0 and 120.0 kcal/mol for (a) O + HCl → OH + Cl (R1), and (b) O + HCl → ClO + H (R2) reactions.

For OH + Cl products (R1 reaction), obvious forward scattering is exhibited in Figure 8(a). The distribution is asymmetric with respect to *θ*_*t*_ = 90*°*, and the peaks are found around *θ*_*t*_ = 0*°*, which indicates that the impact time is short and that direct reaction dominates. Usually, the deep well results in a long-lived reaction intermediate. However, according to the PDDCS_00_ results, direct reaction dominates at high collision energies. The internuclear distances of OH, HCl and ClO (labeled as R_OH, R_HCl, and R_ClO, respectively) as a function of propagation time are shown in Figure 9(a), which gives a proof of the direct reaction. For R1 reaction, the reaction times are short at the three collision energies.

For ClO + H products (R2 reaction), as shown in Figure 8(b), the distribution is observed to be almost backward-forward symmetric with backward scattering being slightly favoured, except for the case of E_col_ = 90.0 kcal/mol. We can deduce that part of the reaction occurs via a long-lived complex and part via direct abstraction of Cl atom, which is also a remarkable conclusion of Ref. [2,5]. Moreover, the peaks locate close to *θ*_*t*_ = 90*°*. H atom (with very slight mass) is one of the products, so the reduced mass of the products is also very small, which causes the products orbital angular momentum ***l***' to be smaller than the products rotational angular momentum ***j***' and the final relative velocity vector ***k***' no longer to be remained in the *xz* plane. Of course, the scattering of the products is still cylindrically symmetric around *z* axis (***k***). So, the separation of H atom and ClO molecule is almost along the direction that perpendicular to the incoming direction of O atom (*i.e.* the direction of ***k***). This results in the dominant population of the products along *θ*_*t*_ = 90*°*. In Figure 9(b), the oscillating structures at the collision energies of 60.0 and 120.0 kcal/mol show that the complexes have long lifetimes compared with the reaction period. Also obviously, the direct reaction occurs at the collision energy of 90.0 kcal/mol.

## Conclusions

For the title reaction, the dynamics of the two product channels through a QCT calculation have been investigated. The RPs, cross sections and branching ratios at high collision energies have been presented and it is found that branching ratio rapidly decreases with the increase of the collision energy. The products are rotationally hot for both R1 and R2 reactions. The alignment and the orientation of the products have been studied, together with the scattering distributions. The HLH channel— OH + Cl (R1 reaction) is the main one as described before. R1 is observed to have essentially isotropic rotational distributions. On the contrary, the HHL one— ClO + H (R2 reaction) is found to be clearly rotationally polarized. The phenomena are probably due to the well structure of the PES and the hyperthermal collision energies. Through PDDCS_00_ results of the O + HCl channel, it is obvious that the impact time is short and that direct reaction dominates. We attribute this to the hyperthermal collisions. However, indirect reactions dominate for the ClO + H channel at the collision energies of 60.0 and 120.0 kcal/mol.

## Competing interests

Are there any non-financial competing interests (political, personal, religious, ideological, academic, intellectual, commercial or any other) to declare in relation to this manuscript? The authors declare that they have no competing interests.

## Authors’ contributions

MG carried out the calculation and drafted the manuscript. MG, HY and YZ analyzed the results. MG, HY and YZ corrected the English expressions. All authors have read and approved the final manuscript.
